# Optimizing enzyme properties to enhance dihydroxyacetone production via methylglyoxal biosensor development

**DOI:** 10.1186/s12934-024-02393-2

**Published:** 2024-05-25

**Authors:** Kaibo Zhang, Mengying Li, Jinsheng Wang, Guozhong Huang, Kang Ma, Jiani Peng, Haoyue Lin, Chunjie Zhang, Honglei Wang, Tao Zhan, Zhe Sun, Xueli Zhang

**Affiliations:** 1 School of Chemistry and Life Science, Changchun University of Technology, Changchun, 130012 Jilin China; 2 Tianjin Institute of Industrial Biotechnology, Chinese Academy of Sciences, Tianjin, 300308 China; 3 College of Biotechnology, Tianjin University of Sciences and Technology, Tianjin, 300457 China; 4 University of Chinese Academy of Sciences, Beijing, 101408 China; 5 National Center of Technology Innovation for Synthetic Biology, Tianjin, 300308 China; 6 Bioengineering College, Chongqing University, Chongqing, 400044 China

**Keywords:** Methylglyoxal biosensor, Dihydroxyacetone, Dihydroxyacetone phosphate dephosphorylase, High-throughput screening, *Escherichia coli*

## Abstract

**Background:**

Dihydroxyacetone (DHA) stands as a crucial chemical material extensively utilized in the cosmetics industry. DHA production through the dephosphorylation of dihydroxyacetone phosphate, an intermediate product of the glycolysis pathway in *Escherichia coli*, presents a prospective alternative for industrial production. However, insights into the pivotal enzyme, dihydroxyacetone phosphate dephosphorylase (HdpA), remain limited for informed engineering. Consequently, the development of an efficient tool for high-throughput screening of HdpA hypermutants becomes imperative.

**Results:**

This study introduces a methylglyoxal biosensor, based on the formaldehyde-responding regulator FrmR, for the selection of HdpA. Initial modifications involved the insertion of the FrmR binding site upstream of the −35 region and into the spacer region between the −10 and −35 regions of the constitutive promoter J23110. Although the hybrid promoter retained constitutive expression, expression of FrmR led to complete repression. The addition of 350 μM methylglyoxal promptly alleviated FrmR inhibition, enhancing promoter activity by more than 40-fold. The methylglyoxal biosensor system exhibited a gradual increase in fluorescence intensity with methylglyoxal concentrations ranging from 10 to 500 μM. Notably, the biosensor system responded to methylglyoxal spontaneously converted from added DHA, facilitating the separation of DHA producing and non-producing strains through flow cytometry sorting. Subsequently, the methylglyoxal biosensor was successfully applied to screen a library of HdpA mutants, identifying two strains harboring specific mutants 267G > T and D110G/G151C that showed improved DHA production by 68% and 114%, respectively. Expressing of these two HdpA mutants directly in a DHA-producing strain also increased DHA production from 1.45 to 1.92 and 2.29 g/L, respectively, demonstrating the enhanced enzyme properties of the HdpA mutants.

**Conclusions:**

The methylglyoxal biosensor offers a novel strategy for constructing genetically encoded biosensors and serves as a robust platform for indirectly determining DHA levels by responding to methylglyoxal. This property enables efficiently screening of HdpA hypermutants to enhance DHA production.

**Supplementary Information:**

The online version contains supplementary material available at 10.1186/s12934-024-02393-2.

## Introduction

Dihydroxyacetone, characterized by its simplicity as the most basic ketose, featuring a ketone functional group and two hydroxyl groups, finds wide-ranging applications in diverse chemical reactions. As a crucial chemical compound, its primary application lies in the cosmetics industry, where it induces the formation of a brown pigment through reactions with amino acids on the skin’s surface. This reaction mimics a tan and offers a viable alternative to excessive UV exposure [1]. The synthesis of DHA can be achieved through chemical means or microbial transformation. However, the associated environmental concerns and heightened costs in chemical synthesis have led to the DHA production via a whole-cell catalytic platform, employing glycerol as a substrate [2]. Glycerol, sourced from the hydrolysis of fats and oils or the byproduct of biodiesel production, undergoes one-step oxidative catalysis by glycerol dehydrogenase to generate DHA directly [3].

*Gluconobacter oxydans* is the predominant bacterium in industrial DHA production due to its exceptional glycerol-oxidizing capabilities. The employment of membrane-bound glycerol dehydrogenase facilitates the transformation of glycerol to DHA within the periplasmic space without entering the cytoplasm [4]. However, drawbacks persist, such as potential deviation of glycerol to the cytoplasm, entering metabolic pathways and reducing glycerol conversion rates [1]. Additionally, elevated glycerol concentrations may hinder the growth of *G. oxydans* [5]. Consequently, optimization strategies, including bioprocess optimization [6–8], cell immobilization [2, 9, 10], and strain engineering [11, 12], becomes imperative. Despite the establishment of gene overexpression, gene knockout, and CRISPR/Cas repression systems in *G. oxydans* [13–15], tools for genetic engineering remain limited compared to classic microorganisms.

In contrast to *G. oxydans*’ glycerol biocatalysis, the redirection of central metabolism in *Saccharomyces cerevisiae* for DHA production involves the expression of NAD^+^-dependent glycerol dehydrogenase, utilizing glucose as the carbon source [16]. Furthermore, the identification of a dihydroxyacetone phosphate dephosphorylase (HdpA) in *Corynebacterium glutamicum* revealed its capacity to catalyze the dephosphorylation of dihydroxyacetone phosphate (DHAP), an intermediate within the glycolysis pathway, resulting in cytoplasmic DHA production [17]. Heterologous expression of HdpA in *Escherichia coli* and *Klebsiella pneumoniae* enables DHA synthesis [18, 19]. In comparison to whole-cell catalysis by *G. oxydans*, this innovative method employs glucose as carbon source, a preference shared by most bacteria, thereby rendering the fermentation process more convenient.

In the pursuit of enhancing Dihydroxyacetone production, strategies involve amplifying the supply of the DHA precursor, DHAP, by the deletion of triose phosphate isomerase (*tpiA*) and methylglyoxal synthase (*mgsA*) [18, 19]. Simultaneously, inhibiting DHA phosphorylation through the deletion of dihydroxyacetone kinase (*dhaK*) improved DHA production [16]. However, despite these advancements, the exploration of the key enzyme HdpA for DHA synthesis remains limited, and avenues for enhancing its enzyme activity remain unexplored. Furthermore, DHA may undergo spontaneously conversion to methylglyoxal (MG) [20], initiating reaction with amino acids in proteins and causing damage to DNA. Consequently, detecting methylglyoxal levels in vivo may provide an indirect approach to determine the DHA concentrations.

In the realms of enzyme engineering and metabolic engineering, biosensors have proven as pivotal tools for the high-throughput screening of target enzymes and dynamic regulation of gene expression through the detection of specific metabolites [21–23]. Although amperometric biosensors, constructed by immobilizing galactose oxidase on specific films, have been developed for quantifying concentrations of DHA and galactose [24, 25], their lack of DHA specificity and a detection limit below the industrial threshold underscore the need for more refined methodologies. The exploration of genetically encoded biosensors for in vivo DHA detection remains unexplored. Notably, the formaldehyde-sensing regulator FrmR has been identified as repressing gene transcription through binding to an inverted repeat sequence (ATACN_9_GTAT). Its repression is relieved upon formaldehyde reacting to form inter-molecular methylene bridges, which subsequently disrupt its binding affinity [26, 27]. This discovery presents a potential route for indirectly detecting DHA by sensing methylglyoxal, the product of DHA conversion. While a previously reported electrochemical biosensor for methylglyoxal exists [28], its scope is limited to detecting methylglyoxal dissolved in solution, rendering it incapable of in vivo detection.

In this study, we focused on harnessing the formaldehyde-sensing regulator FrmR to develop a methylglyoxal biosensor, effectively representing DHA concentration in vivo. To achieve this, the FrmR binding sequence was strategically inserted into several constitutive promoters in *E. coli*, creating a hybrid promoter that is repressed by FrmR and derepressed in the presence of methylglyoxal. The resulting methylglyoxal biosensor was then applied to screen HdpA mutants, leading to the identification of two mutants with improved DHA production. This study demonstrates the effectiveness of the methylglyoxal biosensor in enhancing enzyme property and ultimately boosting DHA production.

## Results

### Engineering of *E. coli* for DHA production from glucose

**Fig. 1:**
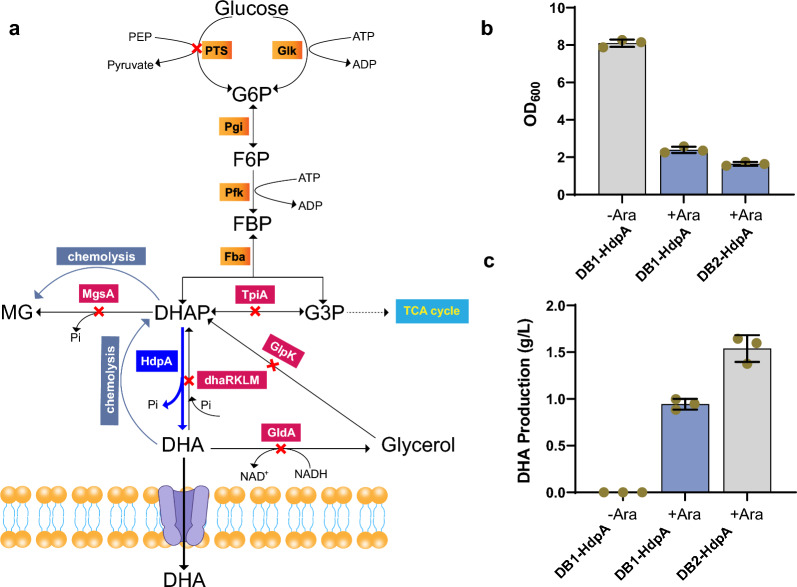
Construction of *E. coli* strains for DHA production from glucose. **a** Schematic representation of the DHA synthesis pathway from glucose and chemolysis, accompanied by genetic modifications implemented to enhance DHA production in *E. coli*. *ptsHI-crr*, glucose phosphotransferase system; *dhaRKLM*, dihydroxyacetone kinase operon; *glpK*, glycerol kinase; *hdpA*, dihydroxyacetone phosphate dephosphorylase; *tpiA*, triose-phosphate isomerase; *mgsA*, methylglyoxal synthase; *gldA*, glycerol dehydrogenase. Comparative analysis of OD_600_ (**b**) and DHA production (**c**) for strains DB1-HdpA and DB2-HdpA. The addition of 2% arabinose for HdpA expression induction is denoted as “+Ara”, while its absence is represented as “−Ara”. Three biological replicates were performed and applied to calculate standard deviation

To establish the DHA production strain, we initiated the process by eliminating the glucose phosphotransferase system, achieved through the targeted knockout of *ptsHI-crr*, which aimed to curtail ATP expenditure during glucose transport into the cytoplasm. Subsequently, the *dhaRKLM* operon was deleted to prevent phosphorylation of the synthesized DHA. Further refinement involved the deletion of the *glpK* gene responsible for glycerol kinase, thereby impeding potential glycerol metabolism (Fig. 1A). The resultant strain, denoted as DB1, formed the foundation for subsequent modifications. Concomitantly, overexpression of the *hdpA* gene, encoding dihydroxyacetone phosphate dephosphorylase from *C. glutamicum*, yielded DB1-HdpA, capable of converting DHAP to DHA. Cultivating DB1-HdpA in shake flasks with M9 medium supplemented with glucose resulted in a DHA production of 0.94 g/L after a 72-h fermentation period (Fig. 1B, C).

To further enhance DHA production, we targeted the triose-phosphate isomerase gene *tpiA* and methylglyoxal synthase gene *mgsA* for disruption, thereby augmenting the availability of the precursor DHAP. Additionally, the glycerol dehydrogenase gene *gldA* was knocked out to mitigate DHA consumption and generate the strain DB2 (Fig. 1A). Strain DB2, following *hdpA* expression (DB2-HdpA), exhibited a 63% increase in DHA production, elevating it from 0.94 to 1.54 g/L compared with DB1-HdpA (Fig. 1B, C). Although DB2-HdpA demonstrated the ability to accumulate DHA, the yield remains suboptimal for industrial applications. Beyond the outlined strategies, a pivotal avenue involves the engineering of the central enzyme HdpA to enhance DHAP transformation. Leveraging genetically encoded biosensors emerges as a fitting strategy to achieve this objective.

### Design and construction of methylglyoxal biosensor

**Fig. 2:**
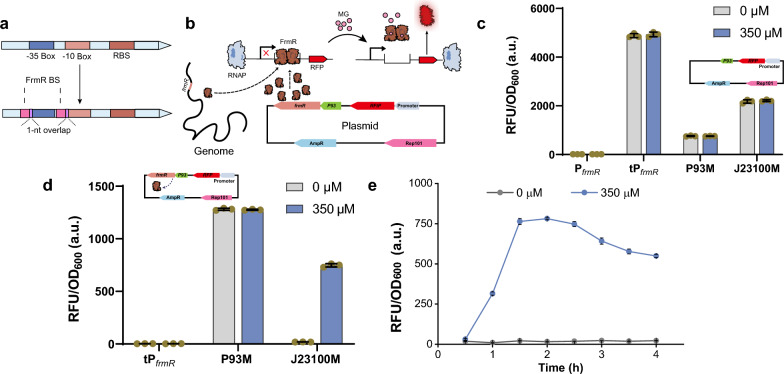
Design and evaluation of the methylglyoxal biosensor.** a** Schematic illustration of the strategy employed for the design of hybrid promoters susceptible to FrmR-mediated repression. Pink bar, FrmR binding site; blue bar, promoter −35 region; Light brown bar, promoter −10 region. **b** Diagram outlining the operational principle of the methylglyoxal biosensor. **c** Fluorescence intensity measurements for strains hosting the hybrid promoters in response to methylglyoxal. P_*frmR*_, *frmR* promoter; tP_*frmR*_, truncated *frmR* promoter; P93M, modified P93 promoter with inserted FrmR binding sites; J23100M, modified J23100 promoter with inserted FrmR binding sites. **d** Impact of FrmR overexpression on the activity of hybrid promoters reacting to methylglyoxal. **e** Dynamic change in fluorescence intensity of the methylglyoxal biosensor corresponding to the duration of methylglyoxal exposure. The experiments were independently conducted with three biological replicates, and standard deviation calculations were applied

There is currently no reported transcriptional regulator specifically responsive to DHA. However, a direct link exists between methylglyoxal and DHA concentrations, as DHA undergoes spontaneous transformation to methylglyoxal in vivo (Fig. 1A). Consequently, we opted to employ the formaldehyde-sensing transcriptional regulator FrmR for the design and construction of the methylglyoxal biosensor. This choice stems from the analogous chemical structures shared by formaldehyde and methylglyoxal, both featuring a carbonyl group. To construct the methylglyoxal biosensor, the FrmR binding site (ATACN_9_GTAT) in *E. coli* was strategically inserted into the promoter regions of two constitutive strong promoters, P93 [29] and J23100 (iGEM Parts Registry), overlapping with the promoter −10 and −35 regions to generate the hybrid P93M and J23100M promoters (Fig. 2A). Notably, the first FrmR binding site, positioned upstream of the −35 region, exhibited a 1-bp overlap, while the second binding site, located in the spacer region between the −10 and −35 regions, displayed a 3-bp overlap with the −10 region. In the absence of methylglyoxal, FrmR was anticipated to inhibit transcription by obstructing key promoter elements. Upon methylglyoxal binding to FrmR, transcriptional activation was expected as FrmR dissociated from the promoters (Fig. 2B).

To assess the functionality of the designed methylglyoxal biosensors, the truncated *frmR* promoter (tP_*frmR*_), a short version of P_*frmR*_ that removed DNA sequences upstream of the FrmR binding sites, and hybrid P93M and J23100M promoters were employed to drive the expression of red fluorescence protein (RFP). However, all promoters consistently drove the same levels of RFP expression, regardless of the presence or absence of methylglyoxal (Fig. 2C and Additional file 1, Fig. S2). This uniform response may be attributed to insufficient levels of FrmR protein derived from single-copy chromosomal expression, resulting in ineffective repression and consequent constitutive expression of RFP in these strains. Consequently, FrmR was further overexpressed by the strong P93 promoter. In both scenarios after FrmR overexpression, with or without 350 μM methylglyoxal, P_*frmR*_ failed to be activated in the presence of the inducer. Conversely, the P93M promoter remained unresponsive to methylglyoxal. The red fluorescence intensity was only induced from the J23100M promoter by 40-fold when methylglyoxal was added (Fig. 2D and Additional file 1: Fig. S2). Further investigation into response kinetics revealed rapid activation of the promoter 1 h after the addition of 350 μM methylglyoxal, reaching peak red fluorescence intensity after 2 h (Fig. 2E). Consequently, we successfully engineered a genetically encoded biosensor that responds to cytoplasmic methylglyoxal.

### Evaluation of methylglyoxal biosensor properties

**Fig. 3:**
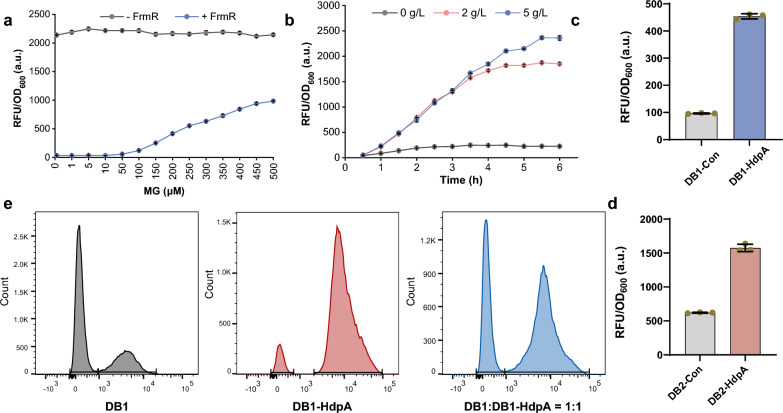
Properties of the methylglyoxal biosensor and DHA monitoring. **a** Profiles depicting the dose dependent fluorescence intensity of the methylglyoxal biosensor in response to varying concentrations of methylglyoxal. −FrmR, absence of FrmR expression from the plasmid; +FrmR, constitutive FrmR expression from the plasmid. **b** Dynamic change in fluorescence intensity of the methylglyoxal biosensor corresponding to the time of DHA treatment. Fluorescence intensity for strains with and without HdpA expression in strains DB1 (**c**) and DB2 (**d**). The presented data were derived from three independent biological replicates. **e** Flow cytometric analysis of the strains DB1-HdpA and DB2-HdpA. Grey, DB1-HdpA; pink, DB2-HdpA; blue, pre-mixture of DB1-HdpA and DB2-HdpA with a ratio of 1:1. A total of 60,000 cells were analyzed for each strain and the mixture of the two strains

Critical properties of biosensors, including substrate sensitivity, affinity range, and signal dynamic range, profoundly impact their screening capabilities and applicability for dynamic gene expression. Our assessment of the constructed methylglyoxal biosensor revealed its remarkable sensitivity, with activation observed at concentrations as low as 10 μM methylglyoxal. The biosensor exhibited a dose-dependent increase in fluorescence intensity with rising methylglyoxal concentrations, plateauing at 500 μM, beyond which cell growth was largely arrested (Fig. 3A). Notably, the fluorescence intensity displayed an approximately 49-fold increase from 10 to 500 μM methylglyoxal, indicating a commendable sensitivity, a broad affinity range and signal dynamic range conducive to enzyme engineering applications. Furthermore, when FrmR was not overexpressed from the plasmid, the J23100M promoter demonstrated constitutive expression, underscoring the indispensable role of FrmR in the methylglyoxal biosensor (Fig. 3A). Subsequently, methylglyoxal added to the medium was replaced with DHA, revealing that the addition of 2 and 5 g/L DHA increased promoter strength by 8.2- and 10.5-fold (Fig. 3B), respectively. Due to the low reactivity and propensity of DHA to cross-link amino acids in a protein, this observation confirmed the responsiveness of the methylglyoxal biosensor to methylglyoxal converted from DHA.

To assess the discriminatory capacity of the methylglyoxal biosensor in distinguishing between strains producing varying DHA levels, we transferred the biosensor system to DB1-HdpA, DB2-HdpA, and their corresponding strains lacking *hdpA* overexpression. After incubation in M9 medium, fluorescence intensities from DB1-HdpA and DB2-HdpA backgrounds were determined and exhibited 4.7- and 2.5-fold compared to control strains without DHA production (Fig. 3C, D). Subsequently, a mixture of strains DB1 and DB1-HdpA harboring the biosensor system underwent flow cytometry, resulting in well-separated cell populations (Fig. 3E). This indicates the potential for high-throughput screening via flow cytometry to select DHA hyper-producing strains based on the methylglyoxal biosensor.

### Enhancing HdpA catalytic activity through methylglyoxal biosensor screening

**Fig. 4:**
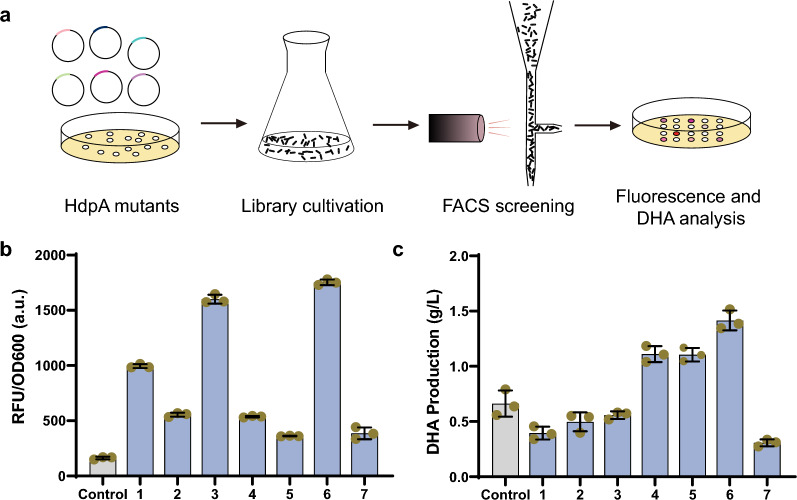
HdpA engineering via methylglyoxal biosensor-based library screening.** a** Schematic diagram illustrating the screening of the HdpA mutant library using the methylglyoxal biosensor in conjunction with flow cytometry. **b** Fluorescence intensity of selected cells after FACS and subsequent cultivation in shake flasks. Each column represents an individual strain. **c** Comparative analysis of DHA production and OD_600_ for strains exhibiting heightened fluorescence intensity. The experiment was independently performed with three biological replicates

Due to the discriminatory capability of the methylglyoxal biosensor among different DHA-producing strains, we employed it to screen for HdpA mutants with enhanced catalytic activity. To achieve this, a library expressing approximately 6.8 million HdpA mutants was generated using error-prone PCR and introduced into the DB1 strain harboring the biosensor system. Following cultivation in M9 medium, library strains underwent fluorescence-activated cell sorting (FACS), and individual cells with the top 0.1% RFP fluorescence were collected (Fig. 4A, Additional file 1: Fig. S1). Subsequent fermentation in shake flasks confirmed that the majority of these collected strains exhibited higher fluorescence intensity compared to DB1-HdpA, aligning well with the FACS sorting outcomes (Fig. 4B). After fermentation, DHA concentrations were measured, revealing that three of seven strains exhibited a 114%, 68% and 68% increase in DHA production compared to wildtype *hdpA* overexpression (Fig. 4C).

These three HdpA mutants yielding higher DHA production were subjected to DNA sequencing, uncovering that the HdpA mutant from the strain (No. 6, Fig. 4C), which displayed the highest DHA production, possessed two-point mutations, D110G/G151C. The HdpA from the other strains (No. 4 and 5, Fig. 4C) had the same synonymous mutation, 267G > T. While the remaining four strains demonstrated higher fluorescence intensities, as indicated by flow cytometric imaging, their DHA productions were comparable or even lower than the HdpA wild type. Upon sequencing the methylglyoxal biosensor system, mutations were identified on FrmR. When the plasmids expressing HdpA mutants were extracted and introduced into the DB1 strain harboring the methylglyoxal biosensor without mutation, the fluorescence intensities reverted to the control level (Additional file 1: Fig. S3), indicating that the FrmR mutants may possess lower or no binding affinity to the J23100M promoter. Consequently, this may render the promoter more susceptible to derepression in the presence of methylglyoxal, leading to higher fluorescence intensities and the generation of false positive results.

### Confirmation of HdpA mutants’ property and enhancement of DHA production

**Fig. 5:**
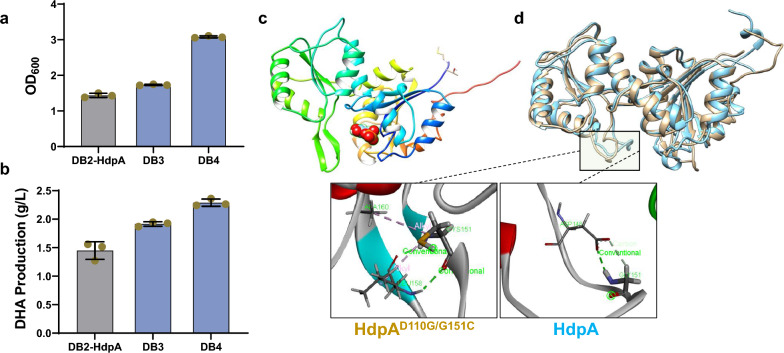
Impact of HdpA mutants expression and structure modeling. Evaluation of OD_600_ (**a**) and DHA production (**b**) for strains DB2 and DB3. The experiments were performed with three biological replicates. **c** DHAP binding in the HdpA catalytic pocket. **d** Structures alignment illustrating the HdpA wild type and D110G/G151C mutant

To ascertain that the augmented DHA production stemmed from the HdpA mutants rather than spontaneous genomic mutations, and to further increase DHA production, the HdpA mutants 267G > T and D110G/G151C, exhibiting superior performance, were introduced into strains DB2, resulting in the development of DHA-producing strain DB3 (DB2-HdpA 267G > T) and DB4 (DB2-HdpA D110G/G151C). Following 72 h fermentation, DB3 and DB4 exhibited higher OD_600_ values, indicative of more efficient utilization of the accumulated intermediate DHAP for detoxification. Moreover, DB3 and DB4 exhibited increased production of DHA to 1.92 and 2.29 g/L, respectively, representing a remarkable increase of 32% and 58% compared to DB2-HdpA (Fig. 5B). This result further underscores the pivotal role of enhanced HdpA activity in improving DHA production.

To investigate the impact of these two mutations on HdpA catalytic activity, we employed the predicted HdpA structure generated by AlphaFold [30, 31] docked with DHAP (Fig. 5C). Structural modeling analysis revealed that the mutant HdpA^D110G/G151C^ demonstrated a novel alkyl-alkyl interaction between C151 and A160. This interaction stabilized the loop formed by amino acids at positions 149–159 [26], thereby preventing the entrapment of the substrate DHAP within the loop (Fig. 5D). Furthermore, the mutation was observed to potentially expand the catalytic pocket, increasing the surface area from 11.75 Å^2^ to 12.15 Å^2^. Additionally, it led to a reduction in the protein’s FullFitness (kcal/mol) score for DHAP, transitioning from −1367.6 to around −1465.2. These potentials may contribute to the enhanced catalytic activity. It’s intriguing that another mutant, which exhibited increased DHA production, harbored a synonymous mutation (267G > T). Even though the codon itself remained unaltered, the mutation may have influenced RNA stability, codon usage, or co-translational folding [32], thereby enhancing the enzyme properties. This examination further underscored the efficacy of the methylglyoxal biosensor in improving HdpA catalytic activity through a systematic high-throughput screening approach.

## Discussion

Genetically encoded biosensors have revolutionized the real-time detection of metabolites, temperature, cell population and other signals within living cells, translating these signals into easily detectable outputs such as fluorescence and color changes [23]. This versatility has found widespread application in metabolic engineering and synthetic biology, encompassing enzyme engineering and pathway optimization [22, 33, 34]. Although genetically encoded biosensors have been predominantly sourced from genomes, natural biosensors often exhibit deficiencies such as limited sensitivity, low affinity, and signal dynamic ranges. In the initial phase of this study, we tested the wildtype *frmR* promoter, only to discover that methylglyoxal failed to activate the promoter. Conversely, the truncated *frmR* promoter, still reserving FrmR binding sites, exhibited a loss of repression by FrmR. This observation implies that numerous allosteric transcription regulators and their corresponding natural promoters may not be suitable for metabolic engineering purposes, necessitating further optimization.

An ideal biosensor should exhibit distinct “OFF” and “ON” states, where promoter activity is almost completely inhibited in the "OFF" state and maximally stimulated in the "ON" state. In our approach, we innovatively developed a methylglyoxal biosensor by strategically inserting the FrmR binding site into constitutive promoters, overlapping with crucial promoter −10 and −35 regions. This design efficiently inhibited promoter recognition by RNA polymerase holoenzyme when FrmR bound to the hybrid promoters [26], as exemplified by the functionality of the J23100M promoter. This strategic design provides a novel and rational approach to biosensor development, although it is essential to acknowledge that this strategy may not be universally applicable to all promoters, as evidenced by its ineffectiveness with P93M. It suggests that biosensor construction, therefor, emerges as a complicated process requiring iterative refinement to meet the essential properties for strain engineering.

The constructed methylglyoxal biosensor exhibits a broad substrate affinity range (10–500 μM) and a significant signal dynamic range (> 30-fold). These features empower the biosensor to effectively quantify the catalytic activities of HdpA mutants, offering an indirect way to measure the DHA levels in a high-throughput fashion when combined with a flow cytometer. Besides DHA, DHAP can also be catalyzed to generate methylglyoxal by methylglyoxal synthase [35]. Thus, the biosensor’s capability to potentially detect dihydroxyacetone phosphate and its derived products may broaden its utility. However, it is critical to note that FrmR, initially identified as a formaldehyde biosensor [26, 27], may not specifically respond to methylglyoxal. Consequently, careful comparison of biosensor output signals in strains with different genetic backgrounds is warranted to account for variations in formaldehyde or other inducers due to genetic modifications. Beyond DHA detection, the biosensor could be applied to detect formaldehyde and derivatives [26, 27], extending its potential applications to enzyme engineering, such as methanol dehydrogenase.

Directed evolution of enzymes for desired properties typically involves two essential stages: library generation and screening. While various in vitro and in vivo methods, such as classic error prone PCR, multiplex automated genome engineering (MAGE) [36] and EvolvR [37], facilitate high mutation rates in the gene of interest. Despite the availability of instruments like flow cytometers and droplet microfluidic sorters capable of processing a large number of cells rapidly, there remains a deficiency in high-throughput screening methods. The absence of suitable screening methods poses a challenge for efficient and rapid screening. This is where properly designed biosensors emerge as potent tools for expedited and effective enzyme engineering. Notably, the dihydroxyacetone phosphate dephosphorylase HdpA lacks a crystal structure, and its catalytic mechanism remains unexplored. This dearth of information complicates efforts to enhance enzyme activity through rational methods. The application of saturation mutagenesis on random sites generates a substantial number of mutants, necessitating labor-intensive screening. The implementation of the methylglyoxal biosensor offers a highly efficient approach to engineer HdpA and other enzymes of relevance.

Typically, industrial *G. oxydans* strains produce 20–40 g/L of DHA in shake flasks without fermentation optimization through glycerol catalysis [4]. Comparing DHA production from *G. oxydans* through glycerol oxidation with the *E. coli* strategy utilizing glucose as the carbon source reveals distinct advantages and disadvantages. While the latter offers a new approach with benefits like a higher growth rate, easier genetic manipulation, and a simpler fermentation process, our current DHA production remains low (2.29 g/L), and it suffers from a lower theoretical maximum yield for DHA. The challenge lies in the fact that glucose, in the glycolysis pathway, transforms into equal amounts of DHAP and glyceraldehyde-3-phosphate (GAP), with GAP entering the tricarboxylic acid (TCA) cycle without DHA production. Addressing this limitation, the study proposes dynamically weakening the TCA cycle using the methylglyoxal biosensor to potentially enhance DHA yield, acknowledging the essential role of the TCA cycle for cellular functions.

## Conclusions

In this study, we developed a methylglyoxal biosensor utilizing the formaldehyde-sensing regulator FrmR and the constitutive promoter J23100. This biosensor exhibited a wide methylglyoxal affinity range and a substantial signal dynamic range. This utility of this biosensor extends to the indirect determination of DHA levels by detecting methylglyoxal spontaneously generated from DHA. Consequently, it enables the efficient separation of strains producing varying concentrations of DHA through flow cytometric fluorescence. Leveraging these distinctive properties, the methylglyoxal biosensor facilitated the screening of hypermutants of HdpA, the pivotal enzyme in DHA production, coupled with FACS sorting. The selected HdpA mutants, namely 267G > T and D110G/G151C, exhibited enhanced DHA production by 68% and 114%, respectively. When expressed directly in strain DB2, these two HdpA mutants increased DHA production from 1.45 to 1.92 and 2.29 g/L, demonstrating the enhanced enzyme properties of the HdpA mutants. Overall, our findings highlight the pivotal role of the methylglyoxal biosensor as a screening platform for identifying HdpA hypermutants with enhanced enzyme properties.

## Materials and methods

### *E. coli* strains, growth conditions and plasmid construction

*E. coli* ATCC 8739 served as the initial host for the construction of the DHA producing strain. The strains constructed in this study were cultured in Luria–Bertani (LB) medium (10 g/L tryptone, 5 g/L yeast extract, 10 g/L NaCl) or on LB plates at 37 °C. When necessary, the medium was supplied with 50 μg/L chloramphenicol or carbenicillin. Target gene deletions were carried out via lambda red homologous recombination using a two-step counter-selection process, as previously reported, without leaving scars [38]. *E. coli* Trans1-T1 (TransGen, Beijing, China) served as the host for plasmids construction. A comprehensive listing of all strains, plasmids, primers, and promoters employed in this study is provided in Additional file 1: Tables S1, S2, S3 and S4.

### Shake flask fermentation, and DHA quantification

For shake flask fermentation, overnight cell cultures in LB medium were transferred to M9 medium (47.8 mM Na_2_HPO_4_, 22.0 mM KH_2_PO_4_, 8.6 mM NaCl, 18.7 mM NH_4_Cl, 0.1 mM CaCl_2_, 1 mM MgSO_4_) containing 20 g/L glucose as the carbon source and 2% arabinose to induce the expression of HdpA. The initiating OD_600_ was adjusted to 0.1, followed by incubation in shake flasks at 37 °C and 250 rpm. In the presence of plasmids, corresponding antibiotics were added to the medium. Fermentation broths were collected at specified time points for the measurement of DHA and glucose concentrations.

The collected fermentation broths underwent centrifugation at 12,000 rpm for 5 min to obtain the supernatant. HPLC (1260 Infinity II, Agilent) coupled with a refractive index detector was employed to determine DHA concentration. For DHA quantification, samples were separated on a Welch Xtimate Sugar-Ca column maintained at 70 °C. The mobile phase, consisting of 0.5 g/L EDTA-Ca in water, was utilized at a flow rate of 0.4 ml/min.

### Methylglyoxal and DHA treatment, and fluorescence measurement

*E. coli* strains harboring the methylglyoxal biosensor system were cultured in LB medium until the OD_600_ reached approximately 1.0 − 1.2. Subsequently, cell cultures were transferred to fresh M9 medium containing 20 g/L glucose with an initial OD_600_ of 0.4. Concurrently, various concentrations of methylglyoxal or DHA were added to the medium, and this time point was designated as 0. The cultures were incubated at 37 °C with a shaking speed of 250 rpm, and samples were collected every 30 min to measure the fluorescence intensity and OD_600_. The collected samples were centrifuged to remove the supernatant, and the cell pellets were resuspended in sterilized water. Fluorescence intensities of the processed samples were measured using a microplate reader (Infinite M200 PRO, Tecan) at excitation and emission wavelengths of 585 nm and 620 nm.

### HdpA library construction

The mutant library of HdpA was generated through error-prone PCR using the QuickMutation Random Mutagenesis Kit (Beyotime). In brief, 1 ng/μl DNA of the codon-optimized *C. glutamicum hdpA* gene served as the PCR template. Following 60 cycles of PCR, the purified PCR product of the hdpA library was assembled with the linear vector in a 3:1 ratio using circular polymerase extension cloning (CPEC) [39]. CPEC products were purified by isopropanol precipitation and transferred to Trans1-T1 competent cells through electroporation. After the addition of 1 ml of SOC medium (0.5% yeast extract, 2% tryptone, 10 mM NaCl, 2.5 mM KCl, 10 mM MgCl2, 10 mM MgSO4, 20 mM Glucose) and incubation at 37 °C and 250 rpm for 1 h, part of the transformation mixture was serially diluted to calculate the total number of transformants. Single colonies grown on LB plates after transformation were sequenced to determine the mutation rates of error-prone PCR. The residual transformation mixture was spread on LB plates with carbenicillin and incubated at 37 °C for 10 h. All cells grown on the plates were pooled, collected with a cell spreader, and transferred to fresh LB medium. After a 2 h incubation, plasmid library extracted and empty plasmid pBAD24 were transferred into DB1 harboring the methylglyoxal biosensor for library screening, respectively.

### Flow cytometry sorting

Strains harboring the HdpA mutant library and the control strain expressing wildtype HdpA were initially cultured in LB medium for 2 h at 37 °C and 250 rpm. Subsequently, they were transferred to M9 medium containing 20 g/L glucose and cultured for another 6 h under 2% arabinose induction. Cells were collected by centrifugation at 3000 rpm for 7 min followed by two washes with PBS (phosphate-buffered saline) buffer. The cell suspension was then diluted to an OD_600_ of 0.025 for analysis using the LSRFortessa X-20 Cell Analyzer (BD Biosciences) with excitation and emission wavelengths set at 566 nm and 610 nm. Further sorting was conducted using the FACSAria Fusion SORP (BD Biosciences), and the cells with top 0.1% of fluorescence intensity were collected on LB plates, where single colonies were incubated until appearance. Subsequently, the collected cells were individually fermented in M9 medium to determine fluorescence intensities and DHA concentrations after 72 h.

### Supplementary Information

**Additional file 1: Fig. S1.** Flow cytometry-based sorting of the HdpA mutant library. **Fig. S2.** Genetic features of plasmids expressing the methylglyoxal biosensor. **Fig. S3.** Verification of fluorescence intensity in FACS-selected cells without improved DHA production. **Table S1.** Strains used in this study. **Table S2.** Plasmids used in this study. **Table S3.** Primers used in this study. **Table S4.** Sequences of promoters used in this study.

## Data Availability

No datasets were generated or analysed during the current study.

## Abbreviations

DHA: Dihydroxyacetone
DHAP: Dihydroxyacetone phosphate
MG: Methylglyoxal
RNAP: RNA polymerase
RFP: Red fluorescence protein
FACS: Fluorescence-activated cell sorting
MAGE: Multiplex automated genome engineering
TCA: Tricarboxylic acid
CPEC: Circular polymerase extension cloning
HPLC: High-performance liquid chromatography
