# Supplementary material for: Optimizing enzyme properties to enhance dihydroxyacetone production via methylglyoxal biosensor development
Source: Microb Cell Fact. 2024 May 25;23:153. doi: 10.1186/s12934-024-02393-2 (PMC11127321; doi:10.1186/s12934-024-02393-2)
Supplement: Supplementary file 1 — Additional file 1: Fig. S1. Flow cytometry-based sorting of the HdpA mutant library. Fig. S2. Genetic features of plasmids expressing the methylglyoxal biosensor. Fig. S3. Verification of fluorescence intensity in FACS-selected cells without improved DHA production. Table S1. Strains used in this study. Table S2. Plasmids used in this study. Table S3. Primers used in this study. Table S4. Sequences of promoters used in this study. [file 12934_2024_2393_MOESM1_ESM.docx]

**Title**

Optimizing enzyme properties to enhance dihydroxyacetone production via methylglyoxal biosensor development

Kaibo Zhang^1, 2†^, Mengying Li^2, 3^, Jinsheng Wang^2, 4^, Guozhong Huang^2, 5^, Kang Ma^2, 3^, Jiani Peng^2, 6^, Haoyue Lin^1, 2^, Chunjie Zhang^1, 2^, Honglei Wang^1, *^, Tao Zhan^2, *^, Zhe Sun^2, 5, *^, Xueli Zhang^2, 5, *^

^1^School of Chemistry and Life Science, Changchun University of Technology, Changchun, Jilin, 130012, China

^2^Tianjin Institute of Industrial Biotechnology, Chinese Academy of Sciences, Tianjin 300308, China

^3^College of Biotechnology, Tianjin University of Sciences and Technology, Tianjin 300457, China

^4^University of Chinese Academy of Sciences, Beijing 101408, China

^5^National Center of Technology Innovation for Synthetic Biology, Tianjin 300308, China

^6^Bioengineering College, Chongqing University, Chongqing 400044, China

^*^Correspondence:

Honglei Wang, Tel: +86-0431-8571-6473, Email: [wanghonglei@ccut.edu.cn](mailto:wanghonglei@ccut.edu.cn)

Tao Zhan, Tel: +86-8486-1946, Email: [elgar@163.com](mailto:elgar@163.com)

Zhe Sun, Tel: +86-2482-8790, Email: [sunzhe@tib.cas.cn](mailto:sunzhe@tib.cas.cn)

Xueli Zhang, Tel: +86-8486-1983, Email: [zhang_xl@tib.cas.cn](mailto:kashlevm@mail.nih.gov)

**Supplementary Information**


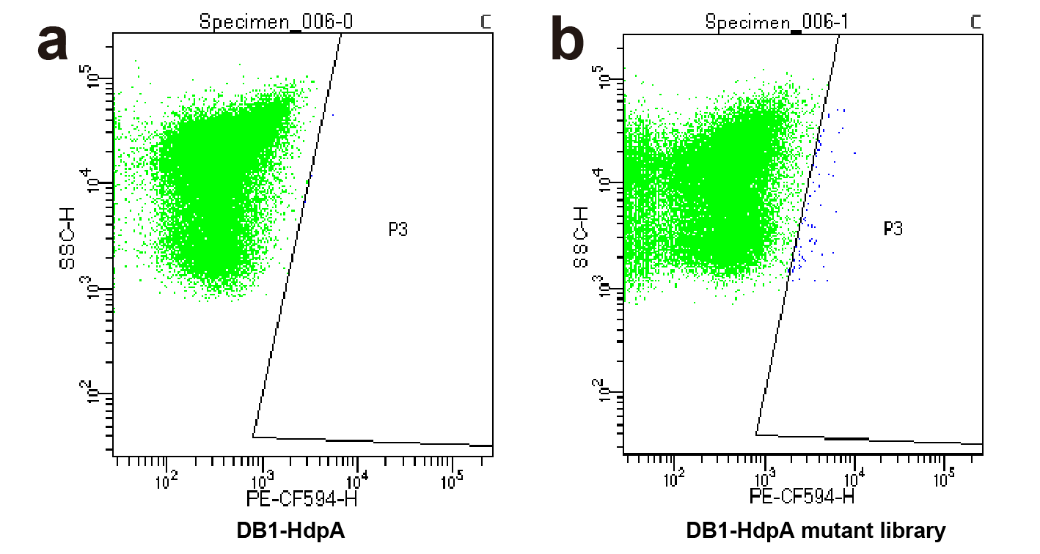


**Supplementary Fig. 1 Flow cytometry-based sorting of the HdpA mutant library. a,** Fluorescence intensity of strain DB1-HdpA, containing the methylglyoxal biosensor, served as the negative control. **b,** Fluorescence intensity of strain DB1 expressing the HdpA mutant library along with the methylglyoxal biosensor. Gating criteria was established using the fluorescence intensity of the negative control.


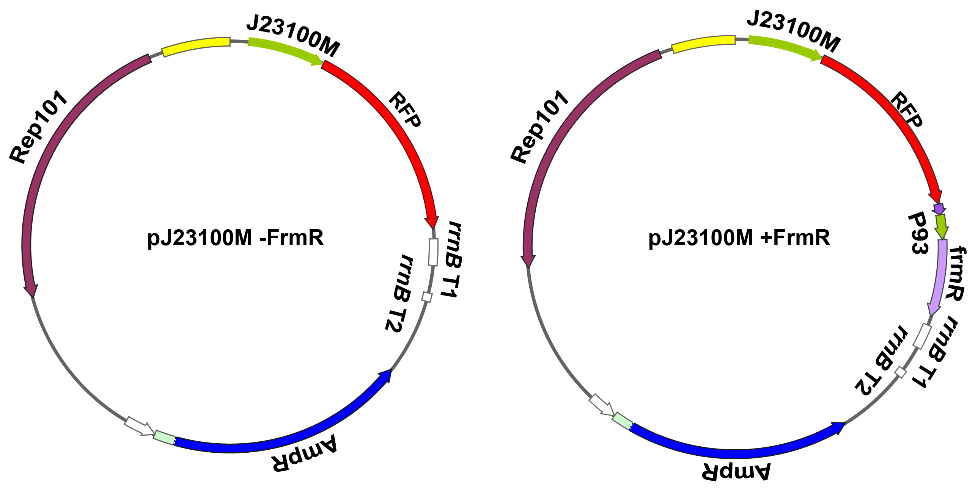


**Supplementary Fig. 2 Genetic features of plasmids expressing the methylglyoxal biosensor.** P93, constitutive strong promoter**;** J23100M, modified J23100 promoter incorporating FrmR binding sites.


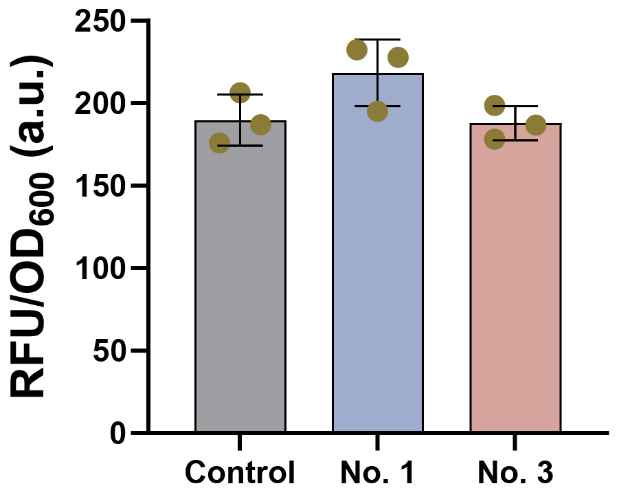


**Supplementary Fig. 3 Verification of fluorescence intensity in FACS-selected cells without improved DHA production.**

| Table S1: Strains used in this study | | |
| --- | --- | --- |
| Name | Relevant genotype and description | Source |
| *E. coli* ATCC 8739 | Wild type | Lab collection |
| *Trans1-T1* | Fφ80 (*lacZ*) ΔM15 Δ*lacX*74 *hsdR* (rk-,mk+) Δ*recA*1398 *endA* *tonA* | TransGen Biotech |
| MG1655 | Wild type | Lab collection |
| DB1 | *E.coli* ATCC 8739 Δ*ptsHI-crr,* Δ*dhaRKLM,* Δ*glpK* | This work |
| DB2 | DB1 Δ*mgsA,* Δ*tpiA,* Δ*gldA* | This work |
| DB3 | The fourth HdpA mutant (267G>T) expressed in DB2 | This work |
| DB4 | The sixth HdpA mutant (D110G/G151C) expressed in DB2 | This work |

| Table S2: Plasmids used in this study | | |
| --- | --- | --- |
| Name | Characteristic | Source |
| pBAD24 | Amp^R^, carries the arabinose inducible promoter | Lab collection |
| pSC105 | Amp^R^ | Lab collection |
| pJ23100-RFP | Amp^R^, the RFP gene was expressed as J23100M on the pSC105 skeleton | This work |
| pFrmR-RFP | Amp^R^, the RFP gene was expressed by the frmR gene itself prompter on the pSC105 skeleton | This work |
| MGp93-RFP | Amp^R^, the RFP gene was expressed as 93 on the pSC105 skeleton | This work |
| spFrmR-RFP | Amp^R^, a plasmid truncated on the promoter of pFrmR-RFP | This work |
| pJ23100-Sensor | Amp^R^, the frmR gene was overexpressed with P93 on the basis of pJ23100-RFP | This work |
| spFrmR-Sensor | Amp^R^, the frmR gene was overexpressed with P93 on the basis of spFrmR-RFP | This work |
| MGp93-Sensor | Amp^R^, the frmR gene was overexpressed with P93 on the basis of MGp93-RFP | This work |
| pJ23100-Sensor-cm | Cm^R^, changed the AmpR of pJ23100-Sensor | This work |
| pBAD24-CgHdpA | Amp^R^, the *hdpA* gene is expressed at the polyclonal site after the promoter of pBAD24 | This work |

| Table S3: Primers used in this study | | |
| --- | --- | --- |
| Name | Sequence |  |
| MGp93-F | ATTGACAAGAATACACAACGTTGGTATAATTGAGCCCGTATTGTTAGCA | Used for rolling ring amplification of MGp93-RFP plasmid |
| MGp93-R | CCAACGTTGTGTATTCTTGTCAATACCGCCAGAGAGTATATGC |  |
| MGp93-YZ-F | ACTCTCTGGCGGTATTGACAAGAATAC |  |
| J23100-RFP-F | TACCAGTCCTAGGTATAGTGCTAGCAGTAGAGAAAGAGGAGAAATACTAGATGGCGAGTAGCGAAGACGT | Used for rolling ring amplification of pJ23100--RFP plasmid |
| J23100-RFP-R | ACTGCTAGCACTATACCTAGGACTGGTATAGCCGTCAATACCGCCAGAGAGTATATGCGCCTGTAGTGCCATTTACC |  |
| J23100-YZ-F | TACCAGTCCTAGGTATAGTGCTAGCAGT |  |
| pSC-P93-F | GGGTAAATGGCACTACAGGCGCTTATCTCTGGCGGTGTTG | Used to amplify pSC105-RFP vector with P93 |
| p93-RFP-WR | ACGTCTTCGCTACTCGCCATAGCTGTTTCCTGGTTTAAACGTACATGC |  |
| spFrmR-YZ-F | TGGCACTACAGGCGCATATACTATAGG |  |
| pSC103-CPEC-F | CCCCATGCGAGAGTAGGGAACT | Used to amplify pSC105-RFP vector |
| RFP-R | TTAAGCACCGGTGGAGTGACGAC |  |
| frmr-f | GCATGTACGTTTAAACCAGGAAACAGCTATGCCCAGTACTCCGGAAGAGAA | Used to amplify *frmR* gene |
| frmr-r | AGTTCCCTACTCTCGCATGGGGCTATTTAAGATAGGCTCGAACCAGTTCAATAGTG |  |
| frmR-CX-F | GGAAGACGGTGCTCTGAAAGGT |  |
| frmR-YZ-R | GCCGTGCCATACTCCAACAGATC |  |
| pSC105-F | ACGTCTCATTTTCGCCAAAAGTTGG | It is used to amplify pSC105 vector without resistance |
| pSC105-R | TTTTTTTAAGGCAGTTATTGGTGCCCTTAAAC |  |
| pSC105-CM-F | GTTTAAGGGCACCAATAACTGCCTTAAAAAAATTACGCCCCGCCCTGC | It is used to amplify cm genes that are homologous to pSC105 |
| pSC105-CM-R | CCAACTTTTGGCGAAAATGAGACGTTGATCGGCACGTAAGAGGTTCCAA |  |
| pBAD24-F | TGATGACCCGGGAAGCTTGG | It is used to amplify pBAD24 vector with arabinose-induced promoter |
| pBAD24-R | CTCTGAATGGCGGGAGTATGAAAAGT |  |
| CgHdpA-CPEC-F | GCTAGCAGGAGGAATTCGCCAATGACAGTAAACATATCATACCTAACTGATATGGATGG | It is used to amplify *CgHdpA* genes that are homologous to pSC105 |
| pBAD-CPEC-R | TGGCGAATTCCTCCTGCTAGC |  |
| pBAD24-CX-F | TATCGCCAGCAGCTCCGAAT |  |
| pBAD24-CX-R | AGTTCCCTACTCTCGCATGGG |  |
| CgHdpA-YZ-R | CGCGCTGGTCCAAATACGTTC |  |
| CgHdpA-CX-F | CATGGTTCTGACCAACAACAGCATCT |  |
| FrmR-YZ-F | AGATCGCTGCCGTTCGGG |  |
| pBAD24-KF | GCTAGCAGGAGGAATTCGCCATGATGACCCGGGAAGCTTGG |  |
| FrmR-YZ-F | CGCGGCTAATGGGCTGATG |  |
| HdpA-YZ-R | GGTCAGACCGCTTTCACCAACCA |  |
| ptsHIcrr-CS-up | CCGCCAGGCTAGACTTTAGTTCCACAACACTAAACCTATAAGTTGGGGAAATACATGTGACGGAAGATCACTTCGCA | Amplification of *Cat-SacB* genes capable of inactivating the PTS system |
| ptsHIcrr-CS-down | GGCGGAAGCATAAAAAAATGGCGCCGATGGGCGCCATTTTTCACTGCGGCAAGAATTATTTGTTAACTGTTAATTGTCCTTGTTC |  |
| ptsHIcrr-f1 | TGTATTTCCCCAACTTATAGGTTTAGTGTTGTG | It is used to amplify and knock out the fragment of the locus *Cat-SacB* gene |
| ptsHIcrr-r1 | CTATAAGTTGGGGAAATACATTCTTGCCGCAGTGAAAAATGGCG |  |
| ptsHIcrr-YZ-up | TTGATTCAGCCTGTCGGAACTGGTATTTA |  |
| ptsHIcrr-YZ-down | CGGTAATGAAGAAAATCAGGAGATGCAG |  |
| dhaKLM-YZ-up | TATGACATCACCGCAGGCAACAGC |  |
| dhaKLM-YZ-down | CATTTTTTGCGGGCGAGAAGGTG |  |
| dhaKLM-CS-up | GTGCGCCGGAATGAAATACAGATCACTCTGATGTATCAGAAATCGTTTCTGTCTTTGTGACGGAAGATCACTTCGCA | It is used to amplify the *Cat-SacB* gene that can replace the dhaKLM site |
| dhaKLM-CS-down | TTAGTGCTGAGTAAATTGCCGGATGACATCAGAACGATGCCATCCGAACAGTGGCTTATTTGTTAACTGTTAATTGTCCTTGTTC |  |
| dhaKLM-f1 | GAAATCGTTTCTGTCTTGCCACTGTTCGGATGGCATCG | It is used to amplify and knock out the fragment of the locus *Cat-SacB* gene |
| dhaKLM-r1 | AAGACAGAAACGATTTCTGATACATCAGAG |  |
| glpK-YZ-up | CCTTGCTGATTGGTCTACTGATTGCG |  |
| glpK-YZ-down | AGCATCGGTGCTTCGTCGATTT |  |
| glpK-CS-up | ACAACTCCTTCAGAACAAAAAGCTTCGCTGTAATATGACTACGGGACAATTAAACTGTGACGGAAGATCACTTCGCA | It is used to amplify the *Cat-SacB* gene that can replace the glpK site |
| glpK-CS-down | CCCGCACGTTTCGGGACTACCGGATGCGGCATAAACGCTTCATTCGGCATTTACATTATTTGTTAACTGTTAATTGTCCTTGTTC |  |
| glpK-f1 | CTACGGGACAATTAAACTGTAAATGCCGAATGAAGCGTTTATGC | It is used to amplify and knock out the fragment of the locus *Cat-SacB* gene |
| glpK-r1 | GTTTAATTGTCCCGTAGTCATATTACAGCGAAG |  |
| mgsA-YZ-up | ATCCGCGAAACGCGCTTCAC |  |
| mgsA-YZ-down | CCAGCAAAGGGCAACAGGCG |  |
| mgsA-CS-up | TGCAGCGATAAGTGCTTACAGTAATCTGTAGGAAAGTTAACTACGGATGTACATTTGTGACGGAAGATCACTTCGCA | It is used to amplify the *Cat-SacB* gene that can replace the mgsA site |
| mgsA-CS-down | GTGCCGGTGGCGAGAAAACCGTAAGAAACAGGTGGCGTTTGCCACCTGTGCAATATTATTTGTTAACTGTTAATTGTCCTTGTTC |  |
| mgsA-f1 | AACTACGGATGTACATTTATTGCACAGGTGGCAAACGCC | It is used to amplify and knock out the fragment of the locus *Cat-SacB* gene |
| mgsA-r1 | AATGTACATCCGTAGTTAACTTTCCTACAGATTAC |  |
| tpiA-YZ-up | CCCATTAATGACCAAAACACAAAGCCAGA |  |
| tpiA-YZ-down | GGTTTGCGCGGGCATGAATAC |  |
| tpiA-CS-up | ACCTGCTGCCCTGCGGGGCGGCCATCTTCCTTTATTCGCTTATAAGCGTGGAGAATTAAATGTGACGGAAGATCACTTCGCA | It is used to amplify the *Cat-SacB* gene that can replace the tpiA site |
| tpiA-CS-down | AGGCGAAGAGTTAAGGAAAGTAAGTGCCGGATATGAAATCCGGCACCTGTCAGACTTATTTGTTAACTGTTAATTGTCCTTGTTC |  |
| tpiA-f1 | ATAAGCGTGGAGAATTAAAGTCTGACAGGTGCCGGATTTCATATC | It is used to amplify and knock out the fragment of the locus *Cat-SacB* gene |
| tpiA-r1 | TTTAATTCTCCACGCTTATAAGCGAATAAAGGAAGA |  |
| gldA-YZ-up | AGGCGGAGACGGCATTCGTA |  |
| gldA-YZ-down | GTGGAACGGCGGGAGATTTTAAGG |  |
| gldA-CS-up | AGGAGCAATTATGGACCGCATTATTCAATCACCGGGTAAATACATCCAGGGCGCTTGTGACGGAAGATCACTTCGCA | It is used to amplify the *Cat-SacB* gene that can replace the gldA site |
| gldA-CS-down | CGCATCAGGCAATTTTGCGTTCAAACTCCCGGACAAGCCGGGAGTTTGGAGTAGGTTATTTGTTAACTGTTAATTGTCCTTGTTC |  |
| gldA-f1 | ATCCAGGGCGCTCCTACTCCAAACTCCCGGCTTGT | It is used to amplify and knock out the fragment of the locus *Cat-SacB* gene |
| gldA-r1 | AGCGCCCTGGATGTATTTACCC |  |

Table S4: Sequences of promoters used in this study

| Name | Sequence |
| --- | --- |
| P*_frmR_* | TTCCTTCTGCCGCCCGCTATCCGGGGCGGCCTTCCCTGCCGATTAGCCCCCCCCCCTTTCCTCTTTGTTTTCCGACCACATTCACCGGATAAATTTTATTCTCCAGTGTTATATACTATAGGGGGGTATGCATTGACATATAGAATACCCCCCTATAGTATATTGCATGCAGATGATGAGGTGCGAA |
| P93M | TCTCTGGCGGTATTGACAAGAATACACAACGTTGGTATAATTGAGCCCGTATTGTTAGCATGTACGTTTAAACCAGGAAACAGCT |
| J23100M | TCTCTGGCGGTAttgacggctaTACcagtcctaggtaTagtgctagcAGTAGAGAAAGAGGAGAAATACTAG |
| tP*_frmR_* | ATATACTATAGGGGGGTATGCATTGACATATAGAATACCCCCCTATAGTATATTGCATGCAGATGATGAGGTGCGAA |
| P93 | TTATCTCTGGCGGTGTTGACAAGAGATAACAACGTTGATATAATTGAGCCCGTATTGTTAGCATGTACGTTTAAACCAGGAAACAGCT |
